# Effects of GaN/AlGaN/Sputtered AlN nucleation layers on performance of GaN-based ultraviolet light-emitting diodes

**DOI:** 10.1038/srep44627

**Published:** 2017-03-15

**Authors:** Hongpo Hu, Shengjun Zhou, Xingtong Liu, Yilin Gao, Chengqun Gui, Sheng Liu

**Affiliations:** 1 School of Power and Mechanical Engineering, Wuhan University, Wuhan, 430072, China; 2 Quantum Wafer Inc., Foshan, 528251, China; 3 State Key Laboratory of Mechanical System and Vibration, School of Mechanical Engineering, Shanghai Jiao Tong University, Shanghai, 200240, China

## Abstract

We report on the demonstration of GaN-based ultraviolet light-emitting diodes (UV LEDs) emitting at 375 nm grown on patterned sapphire substrate (PSS) with *in-situ* low temperature GaN/AlGaN nucleation layers (NLs) and *ex-situ* sputtered AlN NL. The threading dislocation (TD) densities in GaN-based UV LEDs with GaN/AlGaN/sputtered AlN NLs were determined by high-resolution X-ray diffraction (XRD) and cross-sectional transmission electron microscopy (TEM), which revealed that the TD density in UV LED with AlGaN NL was the highest, whereas that in UV LED with sputtered AlN NL was the lowest. The light output power (LOP) of UV LED with AlGaN NL was 18.2% higher than that of UV LED with GaN NL owing to a decrease in the absorption of 375 nm UV light in the AlGaN NL with a larger bandgap. Using a sputtered AlN NL instead of the AlGaN NL, the LOP of UV LED was further enhanced by 11.3%, which is attributed to reduced TD density in InGaN/AlInGaN active region. In the sputtered AlN thickness range of 10–25 nm, the LOP of UV LED with 15-nm-thick sputtered AlN NL was the highest, revealing that optimum thickness of the sputtered AlN NL is around 15 nm.

GaN-based ultraviolet light-emitting diodes (UV LEDs) emitting at wavelength as short as 375 nm have attracted considerable attention for a variety of applications such as sterilization, disinfection, water and air purification, biochemistry, and solid-state lighting^1^,^2^,^3^,^4^,^5^. However, due to the lack of mass-produced bulk GaN substrate, GaN-based UV LEDs are generally grown on foreign substrate such as a sapphire substrate by metal-organic chemical vapor deposition (MOCVD) technique^6^. Although light output power (LOP) of GaN-based UV LEDs has been greatly improved^7^, the performance of GaN-based UV LEDs grown on sapphire substrate is still limited by the crystalline quality of GaN epilayers because of large mismatch in lattice constant and thermal expansion coefficient between GaN and sapphire substrate, leading to a high threading dislocations (TDs) density (10^7^–10^10^ cm^−2^) in GaN epilayers^8^,^9^. This high TD density would generate numerous non-radiative recombination centers, thereby deteriorating the optical and electrical properties of GaN-based devices^10^,^11^. Compared with GaN-based blue LEDs, the efficiency of GaN-based UV LEDs with low In concentrations is more sensitive to TD-related non-radiative recombination centers because of the lack of localized states in multiple quantum well (MQW) active region^12^,^13^. Therefore, it is of particular importance to reduce TD density in GaN epilayers for realization of highly efficient UV LEDs^14^.

To overcome the large lattice mismatch between GaN and sapphire, a low temperature GaN or AlN nucleation layer (NL) is commonly introduced prior to growth of GaN epilayers at high temperature^15^,^16^. Furthermore, several techniques, such as epitaxial lateral overgrowth (ELO)^17^,^18^,^19^, pendeoepitaxy^20^, cantilever epitaxy^21^, *in-situ* SiN_*x*_ nanomasks^22^, and patterned sapphire substrate (PSS)^23^,^24^,^25^ have been employed to reduce TD density. It is reported that high-quality GaN epilayers with reduced TD density can be obtained using a sputtered AlN NL on sapphire substrate^26^,^27^,^28^,^29^,^30^. The quality of GaN epilayers is sensitive to the growth of NL. A systematic comparative study of the effects of *in-situ* low temperature GaN/AlGaN NLs and *ex-situ* sputtered AlN NL on crystalline quality, optical and electrical properties of UV LEDs is also not well investigated yet. In addition, crystalline quality of GaN epilayers is also closely correlated with the thickness of NL^31^,^32^. However, effects of the sputtered AlN NL thickness on crystalline quality, optical and electrical properties of UV LEDs is not well understood yet.

In this study, we demonstrate InGaN/AlInGaN UV LEDs grown on PSS with *in-situ* low temperature GaN/AlGaN NLs and *ex-situ* sputtered AlN NL. The growth behaviors of GaN on PSS with GaN/AlGaN/sputtered AlN NLs were comparatively investigated. We show that the sputtered AlN NL can enhance *c*-plane growth and suppress growth of GaN on cone region of PSS, which can effectively eliminate the undesirable GaN islands on the inclined sidewall of PSS and thus suppress the generation of TDs from coalescence of GaN islands. We also show that with AlGaN NL, the LOP of UV LED is 18.2% higher compared to UV LED with GaN NL owing to a decrease in the absorption of 375 nm UV light in the AlGaN NL with a larger bandgap. With sputtered AlN NL instead of AlGaN NL, the LOP of UV LED is further enhanced by 11.3% due to a reduced TD density in InGaN/AlInGaN MQW. We have further demonstrated that the thickness of sputtered AlN NL has a significant influence on crystalline quality, optical and electrical properties of UV LEDs.

## Results and Discussion

### Structural characterization

**Figure 1:**
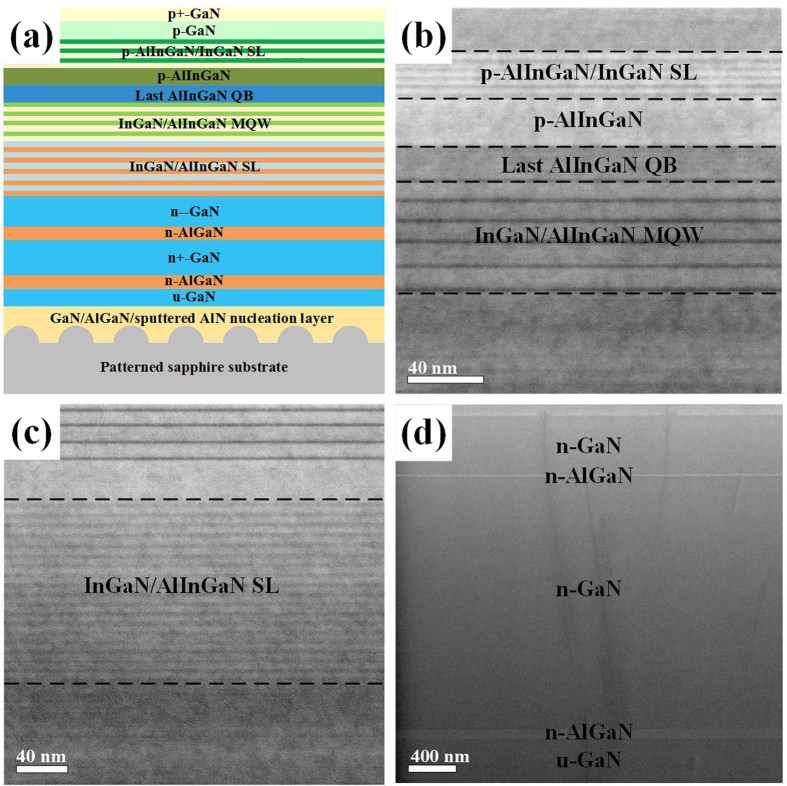
(**a**) Schematic illustration of UV LED structure. (**b**,**c**), and (**d**) Cross-sectional TEM images of UV LED structure.

A schematic illustration of UV LED structure is shown in Fig. 1(a). The UV LEDs were grown on *c*-plane cone-shaped patterned sapphire substrate (PSS) with GaN/AlGaN/sputtered AlN NLs. The epitaxial structures of these UV LEDs are identical except for the NLs. Figure 1(b) shows the cross-sectional TEM image of InGaN/AlInGaN MQW, last AlInGaN quantum barrier layer, p-AlInGaN layer, and five-pair p-AlInGaN/InGaN superlattices. Figure 1(c,d) show eighteen-pair InGaN/AlInGaN superlattices as strain release layer and n-AlGaN interlayers inserted between high temperature GaN layers to improve crystalline quality, respectively.

**Figure 2:**
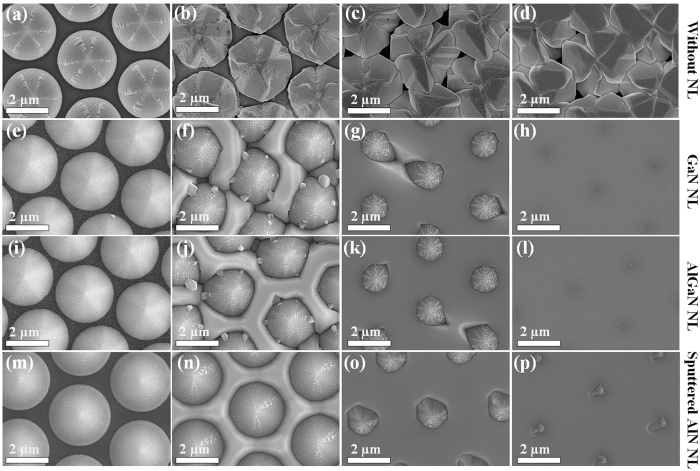
Morphological evolution of GaN grown on PSS with thickness of approximately 30, 300, 700, and 1000 nm, respectively. (**a**–**d**) Top-view SEM images of GaN grown on PSS without NL. (**e**–**h**) Top-view SEM images of GaN grown on PSS with GaN NL. (**i**–**l**) Top-view SEM images of GaN grown on PSS with AlGaN NL. (**m**–**p**) Top-view SEM images of GaN grown on PSS with sputtered AlN NL.

The growth behaviors of the GaN on PSS without NL and with GaN/AlGaN/sputtered AlN NLs for various thickness, namely, 30, 300, 700, and 1000 nm, were observed by scanning electron microscopy (SEM). Figure 2(a–d) show SEM images of the morphological evolution occurring in GaN grown on PSS without NL. Figure 2(e–h) show SEM images of the morphological evolution occurring in GaN grown on PSS with low temperature GaN NL. Figure 2(i–l) show SEM images of the morphological evolution occurring in GaN grown on PSS with low temperature AlGaN NL. Figure 2(m–p) show SEM images of the morphological evolution occurring in GaN grown on PSS with sputtered AlN NL. Without NL, GaN grains mainly grow on the cone region of PSS rather than on the *c*-plane sapphire region as shown in Fig. 2(a–d). During the growth process, the GaN grains do not merge to form bigger GaN grains, leading to the unsuccessfully grown GaN thin film. With the *in-situ* low temperature GaN and AlGaN NLs, the growth of GaN is not only on the *c*-plane sapphire region, but also on the cone region of PSS as shown in Fig. 2(e–h) and Fig. 2(i–l). However, with *ex-situ* sputtered AlN NL, the GaN grow on flat *c*-plane sapphire region rather than on the cone region of PSS, and a two-dimensional lateral growth proceeds favorably for a certain period covering the cone region of PSS as shown in Fig. 2(m–p). On the basis of these observations, it is speculated that the sputtered AlN NL can provide better coverage conditions to enhance *c*-plane growth and suppress growth on the cone region. Reflectance traces and temperature profiles during the growth of GaN on PSS without NL and with GaN/AlGaN/sputtered AlN NLs are shown in Fig. S5.

### Structural characteristics of the GaN/AlGaN/sputtered AlN nucleation layers

**Figure 3:**
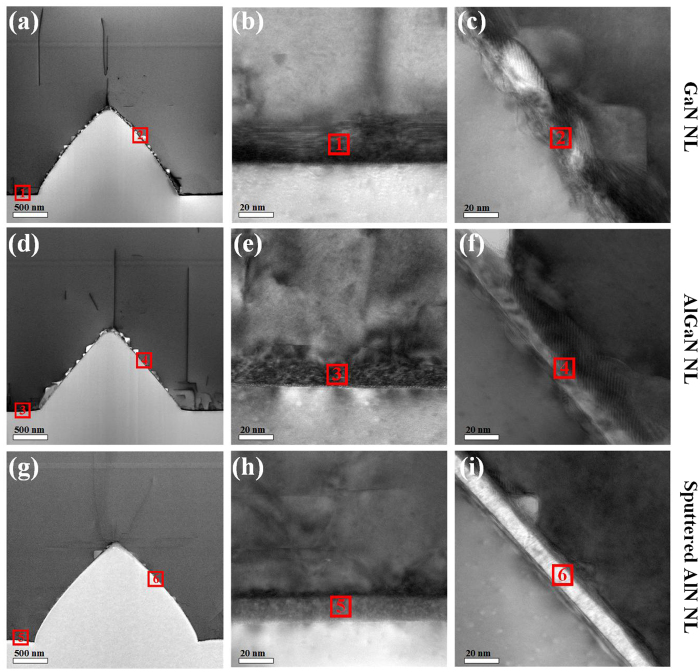
Cross-sectional TEM images of GaN grown on PSS with (**a**) 25-nm-thick GaN NL, (**d**) 25-nm-thick AlGaN NL, and (**g**) 25-nm-thick sputtered AlN NL. 1^*st*^ Row: The magnified TEM images of GaN NL on (**b**) flat *c*-plane sapphire and (**c**) inclined sidewall of PSS. 2^*nd*^ Row: The magnified TEM images of AlGaN NL on (**e**) flat *c*-plane sapphire and (**f**) inclined sidewall of PSS. 3^*rd*^ Row: The magnified TEM images of sputtered AlN NL on (**h**) flat *c*-plane sapphire and (**i**) inclined sidewall of PSS.

Figure 3 shows cross-sectional TEM images of GaN grown on PSS with GaN/AlGaN/sputtered AlN NLs. Figure 3(b,c) show magnified TEM images of GaN NL marked by the red square in Fig. 3(a). Figure 3(e,f) show magnified TEM images of AlGaN NL marked by the red square in Fig. 3(d). Figure 3(h,i) show magnified TEM images of sputtered AlN NL marked by the red square in Fig. 3(g). By contrast, it is observed that the *ex-situ* sputtered AlN NL can provide more uniform thickness across the PSS compared with *in-situ* low temperature GaN/AlGaN NLs. In addition, with the low temperature GaN/AlGaN NLs, there exists a large number of GaN islands on the inclined sidewall of PSS as shown in Fig. 3(a,d), which is consistent with the observation from SEM images of Fig. 2(f) and Fig. 2(j). It is believed that GaN islands on the inclined sidewall of PSS have larger misorientation than those on the flat *c*-plane sapphire, since the GaN islands on the inclined sidewall of PSS nucleate at various crystalline planes rather than single *c*-plane^33^. Therefore, higher TD density will generate when GaN islands on the flat *c*-plane sapphire region merge with GaN islands on the inclined sidewall of PSS^34^,^35^. However, with the sputtered AlN NL, fewer GaN islands appear on the inclined sidewall of PSS, as shown in Fig. 3(g) and Fig. 2(n), which suppresses the generation of TDs through the coalescence of GaN islands from the flat *c*-plane sapphire substrate and inclined sidewall of PSS.

### Threading dislocation (TD) density in the GaN epilayers

**Figure 4:**
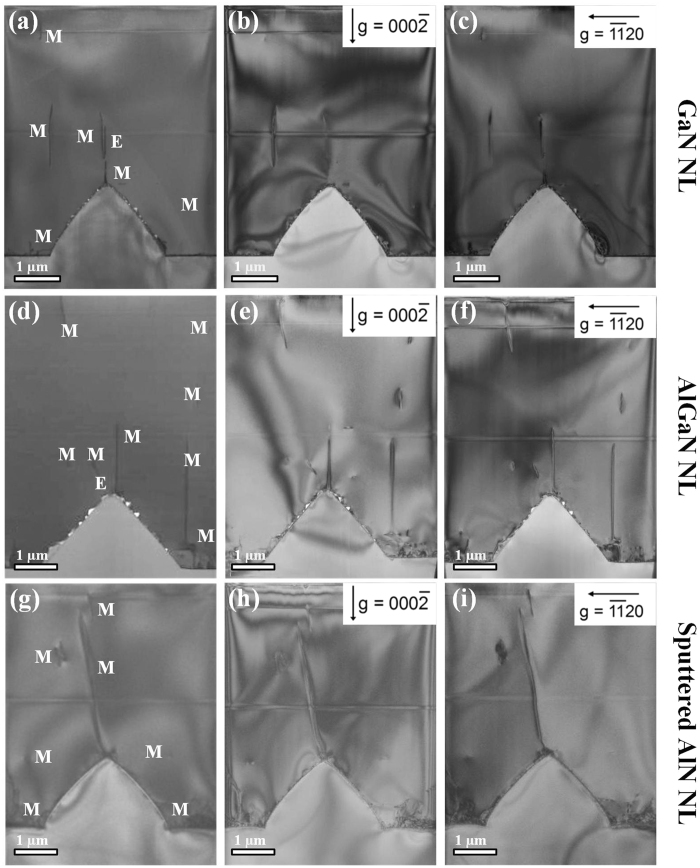
Cross-sectional TEM images of UV LEDs grown on PSS with GaN/AlGaN/sputtered AlN NLs. Bright-field TEM images of UV LED grown on PSS with (**a**) GaN NL, (**d**) AlGaN NL, and (**g**) sputtered AlN NL, zone axis GaN [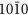]. Bright-filed TEM images of UV LED with (**b**) GaN NL, (**e**) AlGaN NL, and (**h**) sputtered AlN NL when g = 0002. Bright-filed TEM images of UV LED with (**c**) GaN NL, (**f**) AlGaN NL, and (**i**) sputtered AlN NL when g = 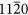.

The cross-sectional TEM images of UV LEDs grown on PSS with GaN/AlGaN/sputtered AlN NLs are shown in Fig. 4. The bright-field cross-sectional TEM images of UV LEDs with GaN/AlGaN/sputtered AlN NLs are demonstrated in Fig. 4(a,d and g), where the type of dislocation including screw (S), edge (E), and mixed (M) is marked. Figure 4(b,e and h) show the bright-field TEM images with g = 0002 for UV LEDs with GaN/AlGaN/sputtered AlN NLs. Figure 4(c,f and i) show bright-field TEM images with g = 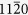 for UV LEDs with GaN/AlGaN/sputtered AlN NLs. The edge and screw dislocations have Burgers vectors (b_*e*_ = 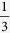 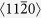 and b_*s*_ = 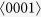, respectively). According to the invisibility criteria^36^, only the screw and mixed dislocations are visible in Fig. 4(b,e and h) when g = 0002; only edge and mixed dislocations are visible in Fig. 4(c,f and i) when g = 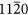
37,38. It is clearly observed that most of these dislocations are visible for both g = 0002 and g = 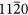 and are thus identified as mixed dislocations. By contrast, it is found that the TD density in UV LED with sputtered AlN NL is the lowest, whereas that in UV LED with AlGaN NL is the highest.

**Figure 5:**
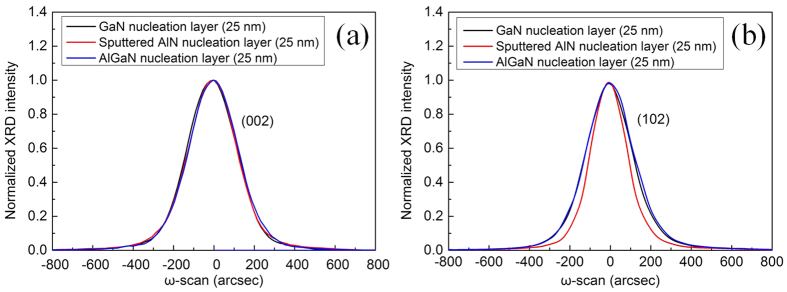
(**a**) Symmetric (002) and (**b**) asymmetric (102) XRD *ω*-scan rocking curves of UV LEDs grown on PSS with GaN/AlGaN/sputtered AlN NLs.

Figure 5 shows symmetric (002) and asymmetric (102) *ω*-scan rocking curves of UV LEDs with GaN/AlGaN/sputtered AlN NLs, respectively. The full widths at half-maximum (FWHMs) of the symmetric (002) rocking curve of UV LEDs with GaN/AlGaN/sputtered AlN NLs are 268.6, 280.5, and 270.4 arcsec, respectively. The FWHMs of the asymmetric (102) rocking curve of UV LEDs with GaN/AlGaN/sputtered AlN NLs are 262, 267, and 208.2 arcsec, respectively. Compared to UV LEDs with low temperature GaN NL, a slight increase of the (002) FWHM and (102) FWHM for UV LED with low temperature AlGaN NL is observed. Furthermore, the (102) FWHM of UV LED with sputtered AlN NL is much lower than that of UV LED with GaN NL. It was previously reported that the FWHMs of symmetric (002) and asymmetric (102) rocking curve are mainly influenced by screw and edge dislocation densities, respectively^39^,^40^. The TD density can be estimated from the FWHMs of X-ray rocking curve *ω*-scan (002) and (102) diffractions using the following equation^41^,^42^.


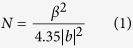


where N is the dislocation density, |*b*| is the magnitude of the Burgers vector, and *β* is the FWHM of the X-ray rocking curve. According to Eq. (1), the screw dislocation densities in UV LEDs with GaN/AlGaN/sputtered AlN NLs are calculated to be 1.449 × 10^8^, 1.581 × 10^8^, and 1.469 × 10^8^ cm^−2^, respectively; the edge dislocation densities in UV LEDs with GaN/AlGaN/sputtered AlN NLs are calculated to be 3.647 × 10^8^, 3.788 × 10^8^, and 2.303 × 10^8^ cm^−2^, respectively. The edge dislocation density in UV LED with sputtered AlN NL shows a dramatic reduction compared to UV LEDs with GaN/AlGaN NLs, whereas the screw dislocation densities in UV LEDs with GaN/AlGaN/sputtered AlN NLs are quite similar. The XRD rocking curves are in good agreement with the results obtained from cross-sectional TEM analyses.

**Figure 6:**
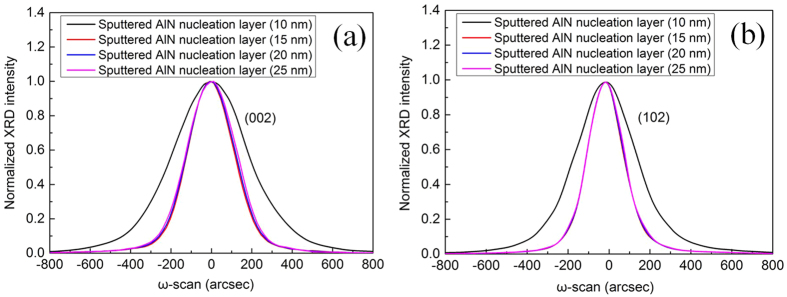
XRD *ω*-rocking scans of (**a**) symmetric (002) and (**b**) asymmetric (102) for UV LEDs grown on PSS with various sputtered AlN NL thicknesses.

Figure 6 shows symmetric (002) and asymmetric (102) *ω*-scan rocking curves of UV LEDs grown on PSS with various sputtered AlN NL thicknesses. When the thickness of sputtered AlN NL is respectively 10, 15, 20, and 25 nm, the FWHMs of GaN (002) rocking curve of UV LEDs are 409.7, 253.7, 260.4, and 270.4 arcsec, respectively; the FWHMs of GaN (102) rocking curve of UV LEDs are 344.6, 202.5, 204.6, and 208.2 arcsec, respectively. As the sputtered AlN NL thickness is decreased to be 10 nm, a steep increase of the (002) FWHM and (102) FWHM is observed, indicating a dramatic degradation of crystalline quality. In the sputtered AlN thickness range of 10–25 nm, the screw dislocation densities in UV LEDs are calculated to be 3.374 × 10^8^, 1.294 × 10^8^, 1.363 × 10^8^, and 1.469 × 10^8^  cm^−2^, respectively; the edge dislocation densities in UV LEDs are calculated to be 6.309 × 10^8^, 2.179 × 10^8^, 2.224 × 10^8^, and 2.303 × 10^8^ cm^−2^, respectively. An appreciable increase of TD density in UV LED is observed as the thickness of sputtered AlN NL is decreased to be 10 nm.

### Optical and electrical characteristics of the InGaN/AlInGaN UV LEDs

**Figure 7:**
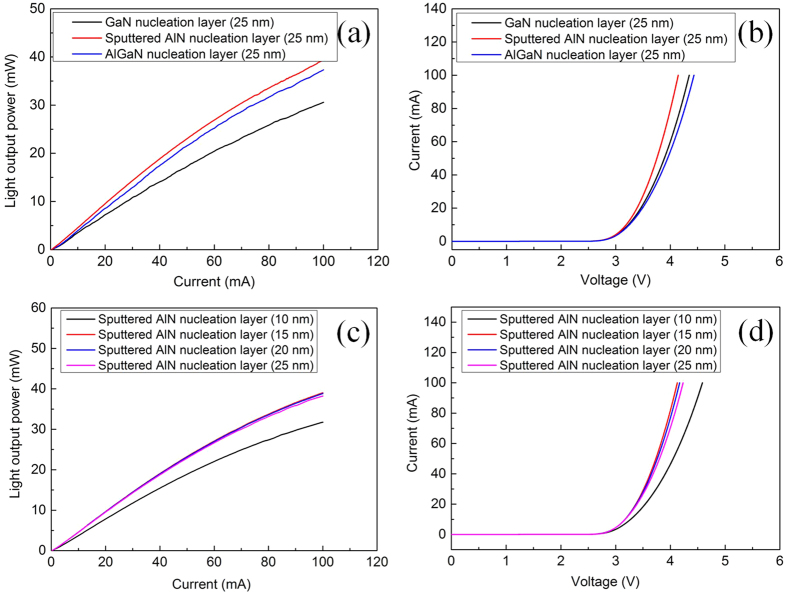
Comparison of optical and electrical characteristics of UV LEDs grown on PSS with different NLs. (**a**) L-I curves of UV LEDs with 25-nm-thick GaN/AlGaN/sputtered AlN NLs. (**b**) I-V curves of UV LEDs with 25-nm-thick GaN/AlGaN/sputtered AlN NLs. (**c**) L-I curves of UV LEDs with various thicknesses of sputtered AlN NL. (**d**) I-V curves of UV LEDs with various thicknesses of sputtered AlN NL.

Electroluminescence (EL) measurements on the UV LED devices were performed using a probe station system. The EL spectra of UV LEDs are obtained at a bias of 20 mA, which reveals the peak wavelength at 375 nm (see Supplementary Fig. S6). Figure 7 shows the LOP–current–voltage (L–I–V) characteristics of UV LEDs with 25-nm-thick GaN/AlGaN/sputtered AlN NLs. At an injection current of 20 mA, the LOPs of UV LEDs with 25-nm-thick GaN/AlGaN/sputtered AlN NLs, as shown in Fig. 7(a), are 7.24, 8.56, and 9.53 mW, respectively; the forward voltages of UV LEDs with 25-nm-thick GaN/AlGaN/sputtered AlN NLs are 3.46, 3.49, and 3.39 V, respectively, as shown in Fig. 7(b). The LOP of UV LED with 25-nm-thick AlGaN NL is 18.2% higher than that of UV LED with 25-nm-thick GaN NL. At 20 mA, the LOPs of UV LEDs with various sputtered AlN NL thicknesses (10, 15, 20, and 25 nm) are 7.81, 9.66, 9.61, and 9.53 mW, respectively, as shown in Fig. 7(c); the forward voltages of UV LEDs with various sputtered AlN NL thicknesses (10, 15, 20, and 25 nm) are 3.54, 3.37, 3.38, and 3.39 V, respectively, as shown in Fig. 7(d). A significant decrease in LOP of UV LED is observed as the thickness of sputtered AlN NL is decreased to be 10 nm.

Although the TD density in UV LED with AlGaN NL is larger than that in UV LED with GaN NL, the LOP of UV LED with AlGaN NL is higher than that of UV LED with GaN NL. It was previously reported by Hasegawa *et al*. that optical absorption coefficient of low temperature GaN NL is markedly larger than that of GaN bulk layer due to massive dislocation defects caused by lattice mismatch between GaN and sapphire^43^. In addition, as the bonding energy of Al-N bond is higher than that of Ga-N bond, the imperfect nature of Ga-N bonds in the low temperature AlGaN is considered to be in favor of forming more Al-N bonds in the AlGaN NL if the gas phase composition (TMAl/(TMGa + TMAl)) is high enough^44^. Here, the gas phase composition (TMAl/(TMGa+TMAl)) for growth of low temperature AlGaN is 0.33, and Al composition in AlGaN NL is estimated to be 0.7. The bandgap of Al_0.7_Ga_0.3_ N NL calculated by Vegard' law is about 5.36 eV, which is much larger than the bandgap of GaN NL. Consequently, the increase in LOP of UV LED with AlGaN NL can be attributed to a decrease in the absorption of 375 nm UV light in the AlGaN NL with a larger bandgap.

It is indicated in Fig. 3 that unfavorable GaN islands on the inclined sidewall of PSS can be effectively eliminated with sputtered AlN nucleation layer, which can suppress the formation of TDs caused by coalescence of GaN islands from the flat *c*-plane sapphire region and inclined sidewall of PSS. Through the results obtained from XRD and TEM, it is also found that the UV LED with sputtered AlN NL shows a dramatic reduction in edge dislocation density in comparison to UV LEDs with GaN/AlGaN NLs. Accordingly, we conclude that the enhancement of LOP of UV LED between sputtered AlN NL and AlGaN NL is dominated by the lower TD density. In the sputtered AlN thickness range of 10–25 nm, the TD density in UV LED with 10-nm-thick sputtered AlN NL is significantly higher than those with thickness of 15, 20, and 25 nm in terms of the results obtained from XRD, thereby leading to a decrease in LOP of UV LED with 10-nm-thick sputtered AlN NL.

## Conclusion

In summary, we have conducted a comparative study of the effects of GaN/AlGaN/sputtered AlN NLs on the crystalline quality, optical and electrical properties of UV LEDs. A series of experiments were carried out to clarify the growth behaviors of GaN on PSS with GaN/AlGaN/sputtered AlN NLs, which demonstrated that sputtered AlN NL can provide a better coverage conditions to enhance *c*-plane growth and suppress growth on cone region of PSS. By replacing GaN NL with AlGaN NL, the LOP of UV LED is improved by 18.2%, which is attributed to a decrease in the absorption of UV light in the transparent AlGaN NL with a larger bandgap. Using a sputtered AlN NL instead of the AlGaN NL, a further improvement of 11.3% in LOP of UV LED is obtained owing to a reduced TD density in InGaN/AlInGaN MQW active region. In the sputtered AlN thickness range of 10–25 nm, the LOP of UV LED with 15-nm-thick sputtered AlN NL is the largest, suggesting that the optimum thickness of the sputtered AlN NL is around 15 nm.

## Methods

### Growth and device fabrication

The cone-shaped PSS was fabricated by combining a thermally reflowed photoresist technique and an inductively coupled plasma (ICP) etching process^45^,^46^. The bottom diameter, height, and spacing of cone-shaped PSS are about 2.7, 1.7, and 0.3 *μ*m, respectively. After preparing the PSS, a thin AlN NL was deposited on the 2-in. *c*-plane (0001) cone-shaped PSS by reactive magnetron sputtering. A 2-in. diameter aluminum layer (99.999% Al) was used as sputtering target. The thin AlN layers with different thicknesses (10, 15, 20, and 25 nm) were deposited on PSS at 650 °C by feeding 120 sccm N_2_, 30 sccm He, and 1 sccm O_2_. By contrast, a 25-nm-thick low temperature GaN NL and a 25-nm-thick low temperature AlGaN NL were also grown on 2-in. *c*-plane (0001) PSS at 530 °C by metal-organic chemical vapor deposition (MOCVD) (see Supplementary Fig. S1) after a standard H_2_ cleaning process at 1050 °C. Trimethylgallium (TMGa), triethylgallium (TEGa), trimetlhylaluminum (TMAl), and trimethylindium (TMIn) were used as group III sources. Ammonia (NH_3_), silane (SiH_4_), and bis(cyclopentadienyl)magnesium (Cp_2_Mg) were used as the group V sources, n-type dopant, and p-type dopant, respectively. The growth process was monitored by laser reflectance with a wavelength of 633 nm. A comparative observation of reflectance traces during the growth of GaN epitaxial layers on different NLs, and the corresponding variation of growth temperature are shown in Supplementary Figs S2 and S3.

The GaN-based UV LEDs, which were grown on both *in-situ* GaN/AlGaN NLs and *ex-situ* sputtered AlN NL in an AIXTRON Crius II_L close coupled showerhead reactor, consisted of a 2.75-*μ*m-thick undoped GaN layer grown at 1025 °C, a 90-nm-thick n-AlGaN layer (n-doping = 2 × 10^18^ cm^−3^) at 970 °C, a 2.23-*μ*m-thick heavily Si-doped n+-GaN layer (n-doping = 1.5 × 10^19^ cm^−3^) at 1025 °C, a 30-nm-thick n-AlGaN layer (n-doping = 9 × 10^18^ cm^−3^) at 1025 °C, a 170-nm-thick lightly doped n-GaN layer (n-doping = 9 × 10^17^ cm^−3^) at 1025 °C, a 144-nm-thick InGaN/AlInGaN superlattices (SLs) at 820 °C, an InGaN/AlInGaN MQW active region including six pairs of 2.7-nm-thick InGaN well layers at 763 °C and 10.3-nm-thick AlInGaN barrier layer at 840 °C, a 19-nm-thick AlInGaN layer (last quantum barrier) at 840 °C, a 28-nm-thick p-AlInGaN layer (p-doping = 1.7 × 10^20^ cm^−3^) at 925 °C, a 26-nm-thick p-AlInGaN/InGaN SLs (p-doping = 1.7 × 10^20^ cm^−3^) at 925 °C, a 50-nm-thick p-GaN layer (p-doping = 6 × 10^19^ cm^−3^) at 950 °C, and a 10-nm-thick heavily Mg-doped p+-GaN layer (p-doping = 1.6 × 10^20^ cm^−3^) at 710 °C.

A transparent conductive indium tin oxide (ITO) layer was used as a p-type ohmic contact layer. A Cr/Pt/Au metallization was deposited as p-type and n-type electrodes, respectively. Finally, the UV LED wafers were diced into chips with size of 203 × 279 *μ*m^2^. We fabricated and characterized six types of UV LEDs, including UV LED with 25-nm-thick low temperature GaN NL, UV LED with 25-nm-thick low temperature AlGaN NL, UV LED with 10-nm-thick sputtered AlN NL, UV LED with 15-nm-thick sputtered AlN NL, UV LED with 20-nm-thick sputtered AlN NL, and UV LED with 25-nm-thick sputtered AlN NL.

### Measurements

The crystalline quality of UV LEDs grown on PSS with GaN/AlGaN/sputtered ALN nucleation layers was characterized by high-resolution X-ray diffraction (XRD), scanning electron microscopy (SEM), and cross-section transmission electron microscopy (TEM). Light output power–current–voltage (L–I–V) characteristics of UV LEDs were measured using an integrating sphere and a semiconductor parameter analyzer (Keysight B2901A).

## Additional Information

**How to cite this article:** Hu, H. *et al*. Effects of GaN/AlGaN/Sputtered AlN nucleation layers on performance of GaN-based ultraviolet light-emitting diodes. *Sci. Rep.* **7**, 44627; doi: 10.1038/srep44627 (2017).

**Publisher's note:** Springer Nature remains neutral with regard to jurisdictional claims in published maps and institutional affiliations.

## Supplementary Material

Supplementary Information

## References

[b1] Khan A., Balakrishnan K. & Katona T. Ultraviolet light-emitting diodes based on group three nitrides. Nat. photon 2, 77–84 (2008).

[b2] Seo T. H. . The role of graphene formed on silver nanowire transparent conductive electrode in ultra-violet light emitting diodes. Sci. Rep. 6, 29464 (2016). 27387274 10.1038/srep29464 PMC4937441

[b3] Kneissl M. . Advances in group III-nitride-based deep UV light-emitting diode technology. Semicond. Sci. Technol. 26, 014036 (2011).

[b4] Hirayama H. . 222–282 nm AlGaN and InAlGaN-based deep-UV LEDs fabricated on high-quality AlN on sapphire. Phys. Status Solidi A. 206, 1176–1182 (2009).

[b5] Dai Q. L., Foley M. E., Breshike C. J., Lita A. & Strouse G. F. Ligand-Passivated Eu:Y_2_O_3_ Nanocrystals as a Phosphor for White Light Emitting Diodes. J. Am. Chem. Soc. 133, 15475–15486 (2011). 21863840 10.1021/ja2039419

[b6] Chang J., Chen D., Xue J. & Dong K. AlGaN-Based Multiple Quantum Well Deep Ultraviolet Light-Emitting Diodes With Polarization Doping. IEEE Photon J. 8, 1600207 (2016).

[b7] Gao N. . Surface-plasmon-enhanced deep-UV light emitting diodes based on AlGaN multi-quantum wells. Sci. Rep. 2, 816 (2012). 23150780 10.1038/srep00816 PMC3495303

[b8] Lester S., Ponce F. A., Craford M. G. & Steigerwald D. A. High dislocation densities in high efficiency GaN-based light-emitting diodes. Appl. Phys. Lett. 66, 1249 (1995).

[b9] Brault J. . Polar and semipolar GaN/Al_0.5_Ga_0.5_N nanostructures for UV light emitters. Semicond. Sci. Technol. 29, 084001 (2014).

[b10] Liu Z. . Efficiency droop in InGaN/GaN multiple-quantum-well blue light-emitting diodes grown on free-standing GaN substrate. Appl. Phys. Lett. 99, 091104 (2011).

[b11] Deng Z. . A novel wavelength-adjusting method in InGaN-based light-emitting diodes. Sci. Rep. 3, 3389 (2013). 24343166 10.1038/srep03389 PMC3865511

[b12] Wang T. . Fabrication of high performance of AlGaN/GaN-based UV light-emitting diodes. J. Cryst. Growth 235, 177–182 (2002).

[b13] Li X. H. . Temperature dependence of the crystalline quality of AlN layer grown on sapphire substrates by metalorganic chemical vapor deposition. J. Cryst. Growth 414, 76–80 (2015).

[b14] Zhao S. . Aluminum nitride nanowire light emitting diodes: Breaking the fundamental bottleneck of deep ultraviolet light sources. Sci. Rep. 5, 8332 (2015). 25684335 10.1038/srep08332 PMC4329565

[b15] Amano H., Sawaki N., Akasaki I. & Toyoda Y. Metalorganic vapor phase epitaxial growth of a high quality GaN film using an AIN buffer layer. Appl. Phys. Lett. 48, 353 (1986).

[b16] Nakamura S., Mukai T. & Senoh M. *In situ* monitoring and Hall measurements of GaN grown with GaN buffer layers. J. Appl. Phys. 71, 5543 (1992).

[b17] Nam O.-H., Bremser M. D., Zheleva T. S. & Davis R. F. Lateral epitaxy of low defect density GaN layers via organometallic vapor phase epitaxy. Appl. Phys. Lett. 71, 2638 (1997).

[b18] Wuu D. S. . Defect reduction and efficiency improvement of near-ultraviolet emitters via laterally overgrown GaN on a GaN/patterned sapphire template. Appl. Phys. Lett. 89, 161105 (2006).

[b19] Xiao M. . A partly-contacted epitaxial lateral overgrowth method applied to GaN material. Sci. Rep. 6, 23842 (2016). 27033154 10.1038/srep23842 PMC4817117

[b20] Linthicum K. . Pendeoepitaxy of gallium nitride thin films. Appl. Phys. Lett. 75, 196 (1999).

[b21] Follstaedt D. M. . Minimizing threading dislocations by redirection during cantilever epitaxial growth of GaN. Appl. Phys. Lett. 81, 2758 (2002).

[b22] Sakai S., Wang T., Morishima Y. & Naoi Y. A new method of reducing dislocation density in GaN layer grown on sapphire substrate by MOVPE. J. Cryst. Growth 221, 334–337 (2000).

[b23] Zhou S. & Liu S. Study on sapphire removal for thin-film LEDs fabrication using CMP and dry etching. Appl. Surf. Sci. 255, 9469–9473 (2009).

[b24] Gao H. . Enhancement of the light output power of InGaN/GaN light-emitting diodes grown on pyramidal patterned sapphire substrates in the micro- and nanoscale. J. Appl. Phys. 103, 014314 (2008).

[b25] Jiang S. . Study on morphology and shape control of volcano patterned sapphire substrates fabricated by imprinting and wet etching. Crystengcomm 17, 3070 (2015).

[b26] Yen C. H. . GaN-Based Light-Emitting Diode With Sputtered AlN Nucleation Layer. IEEE Photonic Tech. Lett. 24, 294–296 (2012).

[b27] Chang L. C., Chen Y. A. & Kuo C. H. Spatial Correlation Between Efficiency and Crystal Structure in GaN-Based Light-Emitting Diodes Prepared on High-Aspect Ratio Patterned Sapphire Substrate With Sputtered AlN Nucleation Layer. IEEE Trans. Electron. Devices 61, 2443–2447 (2014).

[b28] Chiu C. H. . Improved output power of GaN-based ultraviolet light-emitting diodes with sputtered AlN nucleation layer. J. Cryst. Growth 414, 258–262 (2015).

[b29] Chen S. W., Li H. & Lu T. C. Improved performance of GaN based light emitting diodes with *ex-situ* sputtered AlN nucleation layers. AIP Advances 6, 045311 (2016).

[b30] Lee C. Y. . Efficiency improvement of GaN-based ultraviolet light-emitting diodes with reactive plasma deposited AlN nucleation layer on patterned sapphire substrate. Nanoscale Res. Lett. 9, 505 (2014). 25258616 10.1186/1556-276X-9-505 PMC4174280

[b31] Zhang J. C. . The influence of AlN buffer layer thickness on the properties of GaN epilayer. J. Cryst. Growth 268, 24–29 (2004).

[b32] Shang L. . The effect of nucleation layer thickness on the structural evolution and crystal quality of bulk GaN grown by a two-step process on cone-patterned sapphire substrate. J. Cryst. Growth 442, 89–94 (2016).

[b33] Huang X., Liu J., Kong J., Yang H. & Wang H. High-efficiency InGaN-based LEDs grown on patterned sapphire substrates. Opt. Express 19, A949–A955 (2011). 21747566 10.1364/OE.19.00A949

[b34] Wu X. H. . Dislocation generation in GaN heteroepitaxy. J. Cryst. Growth 189, 231–243 (1998).

[b35] Kappers M. J., Moram M. A., Rao D. V. S., Mcaleese C. & Humphreys C. J. Low dislocation density GaN growth on high-temperature AlN buffer layers on (0001) sapphire. J. Cryst. Growth 312, 363–367 (2010).

[b36] Hirsch P. B., Howie A., Nicholson R. B., Pashley D. W. & Whelan M. J. Electron Microscopy of Thin Crystals (Butterworths, London, 1965).

[b37] Gradečak S., Stadelmann P., Wagner V. & Ilegems M. Bending of dislocations in gan during epitaxial lateral overgrowth. Appl. Phys. Lett. 85, 4648–4650 (2004).

[b38] Kim J. . Less strained and more efficient gan light-emitting diodes with embedded silica hollow nanospheres. Sci. Rep. 3, 3201 (2013). 24220259 10.1038/srep03201 PMC3826094

[b39] Yu H., Caliskan D. & Ozbay E. Growth of high crystalline quality semi-insulating GaN layers for high electron mobility transistor applications. J. Appl. Phys. 100, 033501 (2006).

[b40] Heying B., Wu X. H., Keller S. & Li Y. Role of threading dislocation structure on the x-ray diffraction peak widths in epitaxial GaN films. Appl. Phys. Lett. 68, 643–645 (1996).

[b41] Metzger T. . Defect structure of epitaxial GaN films determined by transmission electron microscopy and triple-axis X-ray diffractometry. Philos. Mag. A 77, 1013–1025 (1998).

[b42] Ban K. . Internal Quantum Efficiency of Whole-Composition-Range AlGaN Multiquantum Wells. Appl. Phys. Express 4, 052101 (2011).

[b43] Hasegawa H., Kamimura Y., Edagawa K. & Yonenaga I. Dislocation-related optical absorption in plastically deformed GaN. J. Appl. Phys. 102, 026103 (2007).

[b44] Wang C. & Gong J. Properties of LT-AlGaN films and HT-GaN films using LT-ALGaN buffer layers grown on (0001) sapphire substrates. J. Mater. Sci.: Mater. Electron. 16, 107–110 (2005).

[b45] Park S. H., Jeon H., Sung Y. J. & Yeom G. Y. Refractive sapphire microlenses fabricated by chlorine-based inductively coupled plasma etching. Appl. Opt. 40, 3698–3702 (2001). 18360401 10.1364/ao.40.003698

[b46] Zhou S., Yuan S., Liu S. & Ding H. Improved light output power of LEDs with embedded air voids structure and SiO_2_ current blocking layer. Appl. Surf. Sci. 305, 252–258 (2014).

