# Supplementary material for: Effects of GaN/AlGaN/Sputtered AlN nucleation layers on performance of GaN-based ultraviolet light-emitting diodes
Source: Sci Rep. 2017 Mar 15;7:44627. doi: 10.1038/srep44627 (PMC5353678; doi:10.1038/srep44627)
Supplement: Supplementary Information [file srep44627-s1.pdf]

Effects of GaN/AlGaN/Sputtered AlN nucleation layers on performance of GaN-based ultraviolet light-emitting diodes

Hongpo Hu[1, 3] , Shengjun Zhou[1, 2, *], Xingtong Liu[1], Yilin Gao[1], Chengqun Gui[1], and Sheng Liu[1],

[1]School of Power and Mechanical Engineering, Wuhan University, Wuhan, 430072, China

[2]School of Mechanical Engineering, Shanghai Jiao Tong University, Shanghai, 200240, China

[3]Quantum Wafer Inc., Foshan, 528251, China

*corresponding.zhousj@whu.edu.cn

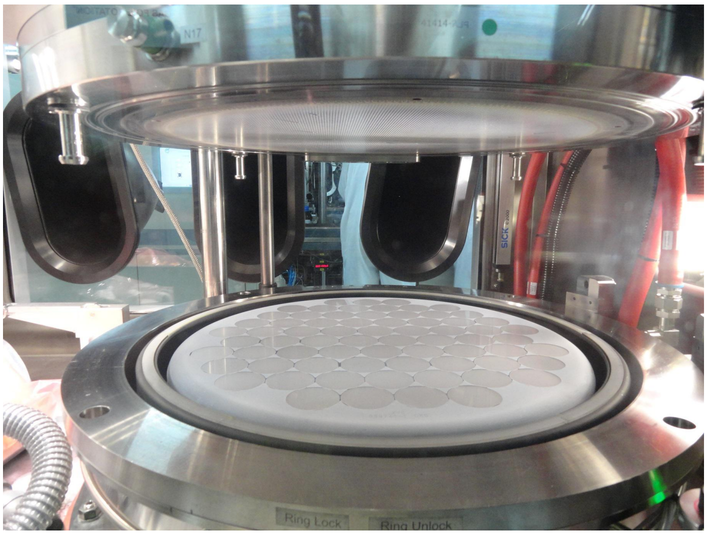

Figure S1. The close coupled showerhead reactor (Crius II_L) for epitaxial growth of UV LEDs.

The UV LEDs with in-situ low temperature GaN/AlN nucleation layers (NLs) and ex-situ sputtered AlN NL were grown in a close coupled showerhead reactor system (AIXTRON Ltd, Crius II_L) as shown in Fig. S1. The growth process is monitored by laser reflectance with a wavelength of 633 nm. Figure S2 shows a comparative observation of reflectance traces during the growth of GaN epilayers on different NLs, and the corresponding variation of growth temperature. As shown in Fig. S2, the growth of GaN epilayers on sputtered AlN NL is different from that on low temperature GaN NL. There is a high temperature bake before the low-temperature GaN NL. Then the growth of GaN epilayers is started as three-dimensional (3D) mode with a low reflectance, followed by the coalescence of GaN islands at a higher temperature. After the coalescence, the GaN epilayers are grown by step-flow mode with an oscillation of reflectance. However, the 3D growth of GaN on sputtered AlN NL is started as the temperature is increased to about 980℃. The temperature and the growth rate then incrases to convert the growth mode from 3D to step-flow mode, resulting in oscillation of reflectance as shown in Fig. S2.

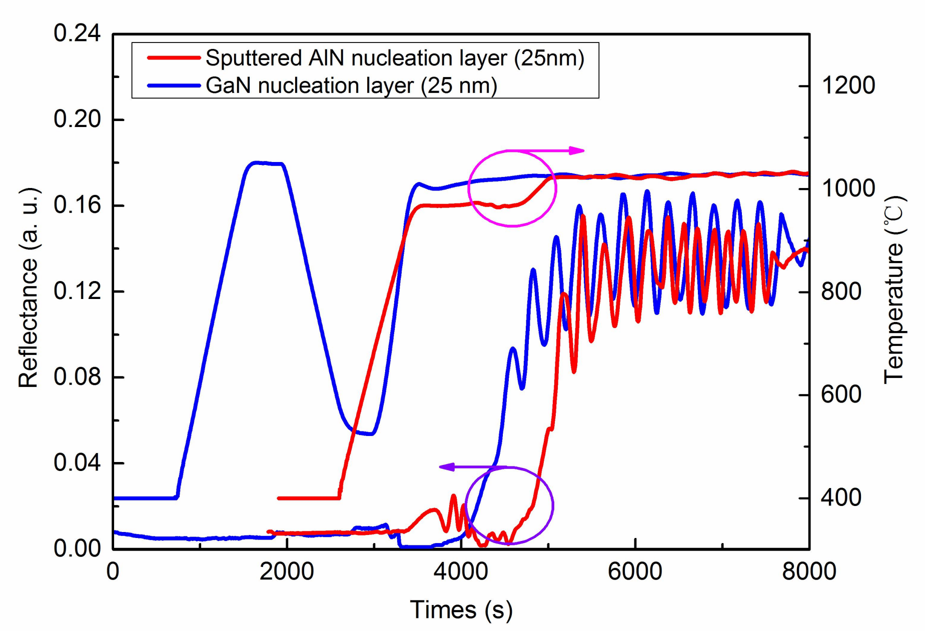

Figure S2. Comparison of reflectance traces and temperature profiles during the growth of GaN on PSS with in-situ low temperature GaN NL and ex-situ sputtered AlN NL.

Figure S3 shows reflectance traces of GaN grown on PSS with various sputtered AlN NL thicknesses. Compared to GaN grown on PSS with 15-nm-thick and 25-nm-thick sputtered AlN NLs, we find that it is more difficult to grow GaN on PSS with 10-nm-thick sputtered AlN NL. The reflectance trace of GaN grown on PSS with 10-nm-thick sputtered AlN NL needs a longer time to convert the growth behavior from 3D to 2D, which is slightly different from those with larger thickness of 15 and 25 nm.

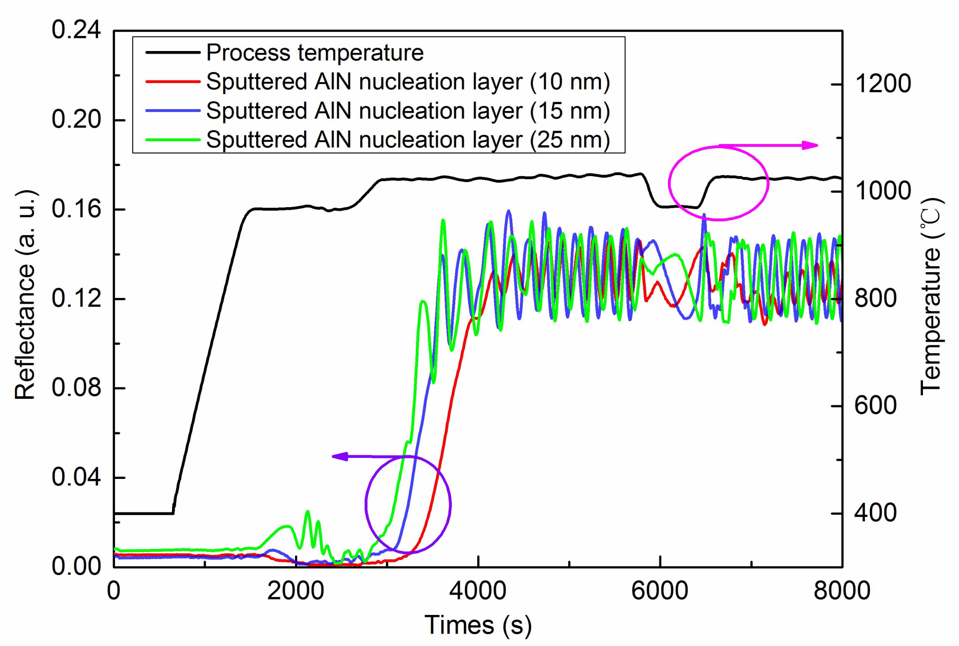

Figure S3. Reflectance traces for GaN grown on PSS with various sputtered AlN NL thicknesses. The temperature profile is also shown for growth process.

GaN grains are deposited on six inclined crystal planes of PSS with horizontal direction of (11-20), and the slant angle of the PSS sidewall is 46.42° as shown in Fig. S4. Therefore, it can be concluded that the GaN grown on PSS without NL is on the plane with Miller indices around (11-26). It was reported that GaN epitaxy on (11-26)-plane sapphire results in (11-20)-plane oriented films. The inclined (11-20)-plane oriented GaN grains cannot merge with each other to form a bigger GaN grain because of different crystal orientations in horizontal plane, leading to unsuccessfully grown GaN thin film.

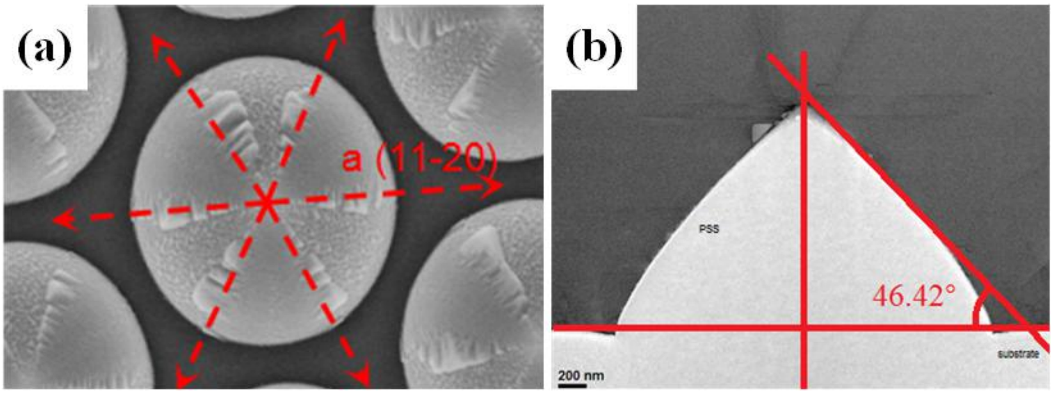

Figure S4. The morphology of PSS. (a) The direction of GaN grown on PSS sidewall. (b) The cross sectional TEM image of GaN on PSS, showing that the slant angle of PSS sidewall is 46.42°.

Reflectance traces and temperature profiles during the growth of GaN on PSS with GaN/AlGaN/sputtered AlN NLs and without NL are shown in Fig. S5, where line (a) is initial time for GaN growth; line (b) is termination time for 30 nm GaN growth; line (c) is termination time for 300 nm GaN growth; line (d) is termination time for 700 nm GaN growth; and line (e) is termination time for 1000 nm GaN growth.

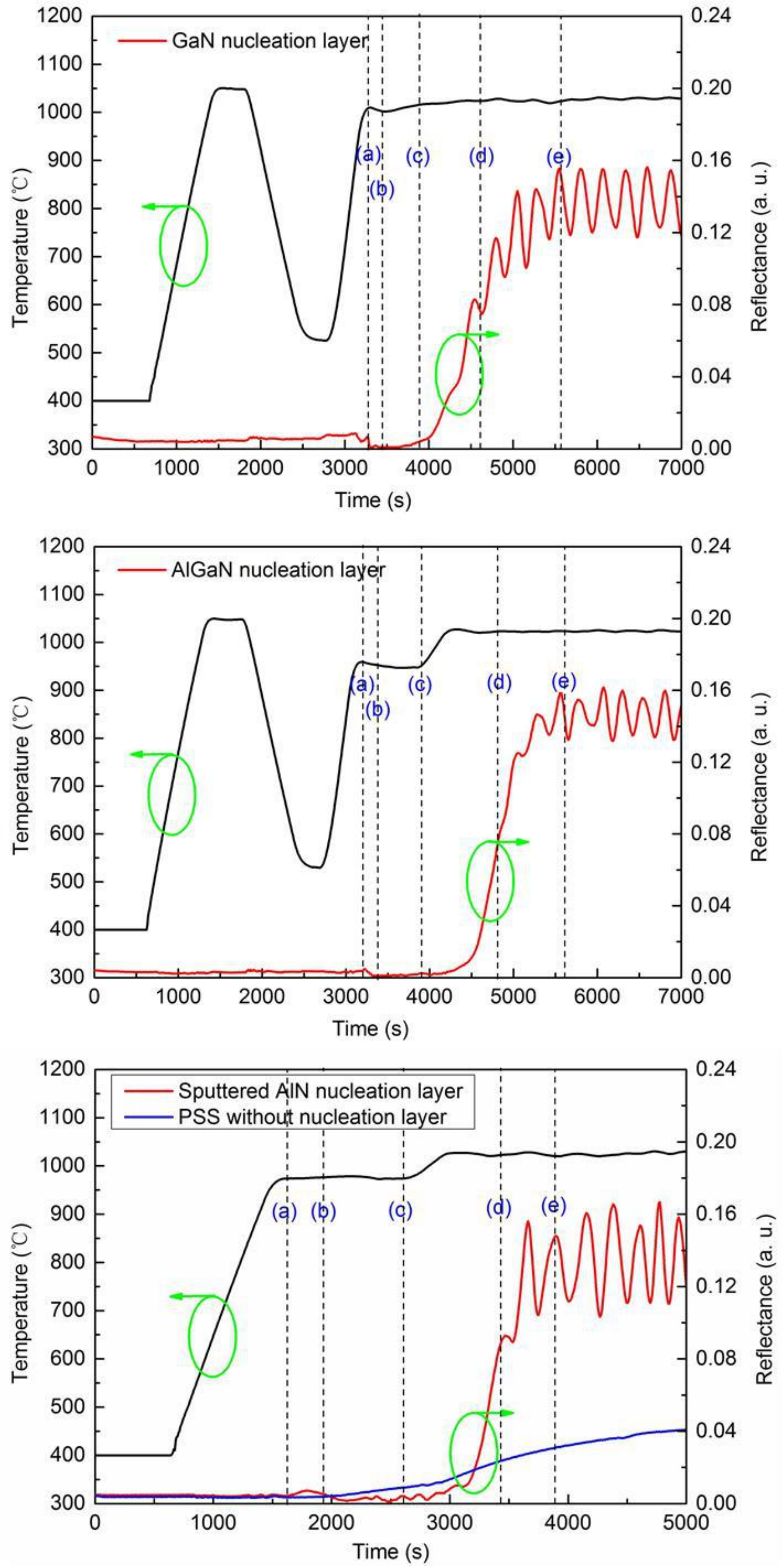

Figure S5. Reflectance traces and temperature profiles during the growth of GaN on PSS with GaN /AlGaN/sputtered AlN NLs and without NL. (a) initial time for GaN growth; (b) termination time for 30 nm GaN growth; (c) termination time for 300 nm GaN growth; (d) termination time for 700 nm GaN growth; (e) termination time for 1000 nm GaN growth.

The peak wavelength of the fabricated UV LEDs was measured by electroluminescence (EL) under a driven current of 20 mA at room temperature. All the LEDs grown on PSS with LT-GaN NL, LT-AlGaN NL and sputtered AlN NL display peak wavelength of emission at about 375 nm, as shown in Fig. S6. Furthermore, the EL peak intensity of the LED grown on sputtered AlN NL is the strongest, whereas the EL peak intensity of the LED grown on LT-GaN NL is the lowest. The enhancement of EL intensity between LT-AlGaN NL and LT-GaN NL is dominated by the lower absorption coefficient of the NL. However, the enhancement of EL intensity between sputtered AlN NL and LT-AlGaN NL is dominated by the lower TD density, since the UV LED structures of the three samples are similar.

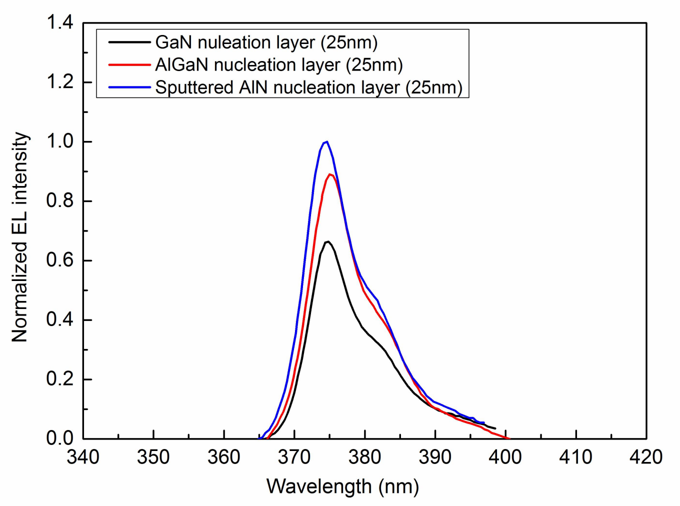

Figure S6. The EL spectra of UV LEDs with GaN/AlGaN/sputtered AlN NLs.
